# Molecular mechanisms by which casein glycomacropeptide maintains internal homeostasis in mice with experimental ulcerative colitis

**DOI:** 10.1371/journal.pone.0181075

**Published:** 2017-07-10

**Authors:** Yongbo Cui, Chenchen Zhu, Zhu Ming, Jiangming Cao, Yali Yan, Pei Zhao, Guangchang Pang, Zixin Deng, Yi Yao, Qingsen Chen

**Affiliations:** 1 School of Basic Medical Sciences, Wuhan University, Wuhan, China; 2 Tianjin Key Laboratory of Food Biotechnology, College of Biotechnology and Food Science, Tianjin University of Commerce, Tianjin, China; 3 Department of Internal Medicine, Yale University School of Medicine, New Haven, Connecticut, United States of America; Indiana University School of Medicine, UNITED STATES

## Abstract

**Objectives:**

The aim of this study was to elucidate the molecular mechanisms by which food-derived casein glycomacropeptide (CGMP) maintains internal homeostasis in the intestinal mucosa and to investigate the effects of CGMP on the intestinal mucosal immunological barrier and related signal transduction pathways.

**Methods:**

In this study, a famoxadone (OXZ)-induced mouse experimental ulcerative colitis (UC) model was built. The experimental UC mice were intragastrically administered milk-derived CGMP for four consecutive days. The molecular mechanisms by which milk-derived CGMP improved and restored the inflammatory status in UC symptoms were elucidated by H&E staining, immunohistochemical staining and western blotting.

**Results:**

The results indicated that CGMP (50 mg/(kg bw·d)) could significantly improve morphological injury to intestinal mucosa in OXZ-induced UC mice to the same extent that did sulfasalazine (SASP, 40 mg/(kg bw·d)), a medicine used to treat UC, in the control group. The study found that CGMP could significantly reduce the expression of Human mucosal addressin cell adhesion molecule-1 (MAdCAM-1), Cluster of differentiation 4 (CD4) and Cluster of differentiation 8 (CD8) in the lamina propria of the intestinal mucosa and significantly stimulate the secretion of sIgA to increase intestinal immunity. Furthermore, CGMP was found to be directly involved in inhibiting the MAPK pathway and activating the TGF-β1/Smad signal transduction cascade, which could maintain immunological regulation of the intestinal mucosa and protect the functions of the intestinal mucosal barrier.

**Conclusions:**

This study elucidated the molecular mechanisms by which CGMP maintained homeostasis of the intestinal mucosa and further confirmed its pharmaceutical value as a food-derived functional component with promising potential for further exploration/utilization.

## Introduction

Inflammatory bowel diseases (IBD), including ulcerative colitis (UC) and Crohn’s disease (CD), are chronic and recurrent intestinal inflammatory diseases [1]. The prevalence and incidence of IBD is increasing worldwide, and IBD greatly diminishes individual quality of life due to pain, vomiting, diarrhea and other socially unacceptable symptoms [2]. Traditional treatment for IBD is focused on using drugs to control active inflammation and regulate the immunological disorder [3]. However, the therapeutically effective drugs for in IBD are frequently associated with toxic side effects and accompanied by a relatively high disease recurrence rate. It has been reported that approximately 2/3 cases of CD patients and 1/3 of UC patients eventually require surgical therapy [4, 5]. Currently, food-derived functional components have driven more and more attention in improving human health/wellness, such as for the treatment of autoimmune diseases [6, 7]. Thus, the development of a natural food-derived, biologically active component as a therapeutic nutritional regimen has become a hot research topic in this field [2, 8].

Currently, many investigators believe that the initiation, development and progression of IBD are caused by disordered immunological regulation in the intestinal mucosa or a defective intestinal mucosal barrier. Certain immunological barrier factors, structural disorder of intestinal flora, and genetic and environmental factors cooperate to drive the disease [9–11]. Although some breakthroughs have been made in studying the pathogenic mechanisms of IBD due to the influence of environmental factors, genetic factors and immunological disorder [12], many aspects of the pathogenic mechanisms of UC and CD remain largely unknown. Immunological disorder is a key factor involved in the pathogenesis of intestinal inflammatory diseases and is related to modified signal transduction pathways in the intestine [13]. Currently, the scientific community has recognized the importance of applying new dietary therapies to the prevention, improvement and therapeutic treatment of autoimmune diseases, which are difficult to cure, and the pathogenic mechanisms are unknown. Thus, for ulcerative colitis, it is necessary to conduct in-depth studies of the immunological barrier and immunological signal transduction pathways in the intestinal mucosa to resolve problems such as IBD that affect human health and longevity [14].

Mitogen/extracellular-signal regulated kinase kinase-1 (MEKK1) is situated in a key position in the mitogen activation protein kinase (MAPK) signal transduction pathway. Extracellular signal-regulated kinase 1/2 (ERK1/2) is activated by phosphorylation via the Ras-Raf-MEKK1 pathway and enters the nuclei where it acts on transcription factors and participates in multiple biological reactions. MEKK1 interacts via its catalytic domain with the upstream mitogen-activated protein kinase-1 (MKK-1) and activates ERK-1 both *in vitro* and *in vivo* [14]. Over-expression of MEKK1 can lead to strong activation of ERK1/2, JNK, p38 and IκB kinase, which are involved in injury processes such as the release of proinflammatory cytokines and apoptosis in intestinal epithelial cells and in the regulation of inflammatory responses [15].

Transforming growth factor-β1 (TGF-β1) is a cytokine belonging to TGF-β superfamily that exerts important roles in the regulation of many physiological and pathological processes such as cell growth and differentiation [16]. The effects of the TGF-β family can be mediated via multiple signal transduction pathways, including the classic Smad signaling pathway. Smads are signaling molecules that are present in the cytoplasm and can directly transduce TGF-β signaling into the nucleus through the cell membrane. Interrelations between the TGF-β1 and Smad signaling pathways could offer beneficial insight for the therapeutic treatment and diagnosis of IBD and the effective amelioration of IBD symptoms [17].

Casein glycomacropeptide (CGMP) is a hydrophilic glycol-peptide discovered in 1965, and its carbohydrate domain is the structural basis that allows CGMP to perform its various biological functions [18]. CGMP is rich in branched-chain amino acids and low in methionine, which makes it ideal for patients suffering from hepatic diseases [19]. Hvas et al. [20] used CGMP as a nutritional therapy to standardly treat patients with active distal UC and found that CGMP was well tolerated and accepted by the patients, and the disease-modifying effect of CGMP was similar to that of the mesalamine dose escalation. Rong et al.[21] fed *Escherichia coli K88*-challenged piglets CGMP as a potential feed additive and found that a diet containing 1% CGMP significantly alleviated the decrease in average daily gain (P < 0.05), increase in pathogenic bacteria amounts in intestinal contents (P < 0.05), and intestinal morphology. Ortega et al. [22] found that milk k-casein-derived CGMP exhibited intestinal anti-inflammatory effects in a lymphocyte-transfer mouse model of colitis by reducing the activity of colonic myeloperoxidase and the percentage of CD4⁺ interferon (IFN)-γ⁺ cells in mesenteric lymph nodes. As a potential nutritional therapeutic regimen, milk-derived CGMP could be a new option for the amelioration, repair and therapeutic treatment of IBD. Therefore, conducting an in-depth study of the molecular mechanisms underlying the therapeutic effects of treatment with milk-derived CGMP for IBD is of high importance and practical significance.

## Materials and methods

### Experimental animals

BALB/c mice (male, specific pathogen-free (SPS) grade, weighing 25 ± 2 g) were purchased from The Center for Experimental Animals of the Academy of Military Medical Sciences, People's Liberation Army of China (Beijing, China). The animal experiments were performed at the Animal Research Centre of TIENS Group Co. Ltd. (Tianjin, China) and passed the National Certification. A total of 100 mice were housed under a specific pathogen-free (SPF) environment at an animal house facility under conditions of constant temperature (21±2°C) and humidity (55±10%) with a 12 h/12 h light/dark cycle. The animals were provided with free access to tap water and routine mouse chow. The mice status was observed daily, including physical activities, defecation, body weight, amount of food intake and spirit. No other activities were observed. At the end of study (or if body weight loss > 15% was observed during the study), the mice were sacrificed by cervical dislocation under general anesthesia with isoflurane (3~4%). None of the mice died spontaneously, and all mice were sacrificed at the end of the study when the colon was removed, dissected and opened lengthwise.

This study was carried out in strict accordance with the recommendations in the Guide for the Care and Use of Laboratory Animals of the National Institutes of Health and Regulations for the Administration of Affairs Concerning Experimental Animals in China (2011). The experimental protocol was approved by the Committee on the Ethics of Animal Experiments of Tianjin University of Commerce, The Center for Experimental Animals of the Academy of Military Medical Sciences, People's Liberation Army of China and Animal Research Centre of TIENS Group Co. Ltd. (Permit Number: SYXK(Jin)2012-0004).

### Reagents

CGMP with a purity of 71% containing 5.6% sialic acid was purchased from Tatua Dairy Company (North Island, New Zealand). This was the highest purity food-derived CGMP that could be purchased from the market. Sulfasalazine (SASP) was purchased from Sanwei Pharmaceuticals Company of Shanghai. Famoxadone (OXZ) was obtained from Sigma. An anti-mouse polyclonal MEKK1 antibody, an anti-mouse β-actin monoclonal antibody, and an anti-mouse polyclonal GAPDH antibody were all purchased from Santa Cruz Biotechnology (Santa Cruz, CA, USA). An anti-mouse Smad3 monoclonal antibody and an anti-mouse Smad-7 monoclonal antibody were obtained from Epitomics Inc. (Burlingame, CA, USA). Horseradish peroxidase (HRP)-labeled goat anti-mouse IgG and HRP-labeled goat anti-rabbit IgG were purchased from Beijing Zhong Shan-Golden Bridge Biological Technology Co., Ltd. (Beijing, China). An anti-mouse polyclonal CD4 antibody, an anti-mouse polyclonal CD8 antibody, an anti-mouse polyclonal MADCAM-1 antibody and a polyclonal IgA antibody were obtained from Beijing Boaosen Biotechnology Ltd. (Beijing, China).

### Experimental UC mouse model

After being adaptively fed for one week, the mice were randomly divided into 5 groups with 20 mice in each group as follows: A) normal control group (represented by “N” in the chart); B) model control group (represented by “M” in the chart); C) CGMP group 1 (50 mg/(kg bw•d), represented by “C1” in the chart); D) CGMP group 2 (200 mg/(kg bw•d), represented by “C2” in the chart); and E) SASP-treatment group (40 mg/(kg bw•d), represented “S” in the chart). A mouse model of UC was established using a method previously described by Frank Heller et al.[23]. The mice in the normal control group were perfused with 0.15 ml of 50% ethanol and used as controls; the mice in the other groups were perfused with 0.15 ml of 1% famoxadone (dissolved in 50% ethanol). After the enema, the mice in CGMP group 1 and CGMP group 2 were intragastrically administered CGMP (50 mg/(kg bw•d) and 200 mg/(kg bw•d)). The mice in the SASP-treated group were intragastrically administered SASP (40 mg/(kg bw•d). The mice in the control and model groups were fed only normal mouse food, and the first day of treatment was recorded. The mice in the remaining groups were intragastrically administered CGMP and SASP at the indicated doses for four consecutive days. Mouse status was observed daily, including physical activities, defecation, body weight, amount of food intake and spirit. The changes in the body weights of all mice were monitored and recorded in detail.

### Histological scoring

The morphological injury indices for the experimental UC mice and the methods for histological scoring have been described previously [24, 25]. Briefly, tissue/cell intestinal sections were fixed in 4% paraformaldehyde and paraffin-embedded. Antigen retrieval was performed with citrate buffer (0.01 M, pH 6.0) and a boiling bath for 15 min. The slides were blocked with endogenous peroxidase by incubation with 3% Hydrogen peroxide for 30 min and blocking buffer (normal goat serum, C-0005) at 37°C for 20 min. The slides were then incubated with anti-MAdCAM1 Polyclonal Antibody, unconjugated 1:200, overnight at 4°C, followed by conjugation to the secondary antibody and DAB staining.

### Immunohistochemistry

On the 4^th^ day after intragastric administration of CGMP, the pathologically altered colon segment sections from the mouse intestine were removed, and fecal residue was washed away with sterile PBS buffer. The intestinal sections were placed into an embedding box and soaked in formaldehyde solution to fix the tissues for 24 h at 4°C. The protein expression levels of CD4, CD8, sIgA and MAdCAM-1 in the intestinal mucosae of the experimental UC mice were detected using a previously described immunohistochemical method [26]. The results were acquired by collecting images of immunohistochemical staining under a microscope. The positively stained areas with brown-yellow color were analyzed from each of the acquired images with a biological image processing system, Image Pro-plus 6.0, to obtain integrated optical density (IOD) values. dx.doi.org/10.17504/protocols.io.h8fb9tn.

### Western blotting

On the 4^th^ day after treatment with CGMP, the pathologically altered intestinal sections from experimental UC mice were removed, washed with sterile PBS buffer to remove fecal residue, and placed into pre-cooled, labeled EP tubes. The amount of intestinal tissue was weighed, and total protein was extracted from the intestinal tissue. Protein concentration was quantified using a BCA Protein Assay Kit. The protein expression levels of MEKK1, Smad3 and Smad7 in the intestinal mucosae of the experimental UC mice were assayed by Western blotting. The gray-scale values for each of the targeted protein bands and the internal control protein band were analyzed using Quantity One software [27]. dx.doi.org/10.17504/protocols.io.h8hb9t6.

### Statistical analyses

Data treatment and one-way ANOVA analysis of the experimental data were conducted with SPSS17.0 statistical analysis software. The results are shown as the mean ± standard deviation (X ± SD). The differences between groups at *P*<0.05 was regarded as significant. The difference between groups at *P*<0.01 was regarded as extremely significant.

## Results

### Treatment with milk-derived CGMP ameliorated abnormal symptoms in experimental UC mice

During the experimental period, the mice in the normal control group exhibited normal eating, physical activity and defecation behaviors. Their hair was glossy, and the changes in their body weights exhibited an increasing trend. The mice in the OXZ-induced UC model group had reduced body weight, food intake and physical activities, loose hair without gloss, loose stool, diarrhea, sticky anal secretions, and hematochezia compared with the normal control group. Compared with mice in the normal control group, the body weights in the OXZ-induced UC model group were dramatically reduced on the second day after the intragastric administration of OXZ (Table 1). The other symptoms were also aggravated concordantly (data not shown). On the 4^th^ day after the administration of OXZ, the body weights of most mice in OXZ-induced UC model group were still reduced, whereas the body weights of a few mice were slightly increased. These mice displayed signs that indicated the beginning of restored eating, physical activities and defecation behaviors, but their hair still lacked gloss, and their body weights still exhibited a decreasing trend compared with their initial body weights. Although the mice in the groups administered CGMP (groups C1 and C2) exhibited symptoms indicating the passage of loose stool and diarrhea in the first two days after treatment, these symptoms were relatively mild compared with the mice in the OXZ-treated group. Starting on the third day after treatment, the eating status of CGMP-treated mice was essentially similar to that of mice in the normal control group. These mice also began to demonstrate reductions in loose hair and diarrhea as well as restored physical activities. The eating, physical activities and defecation behaviors of the mice in the SASP-treated group were not significantly different from those of the mice in CGMP-treated groups. These data indicated that although the four-day experimental period did not adequately reflect the restoration of body weight in mice, treatment with milk-derived CGMP at appropriate doses clearly restored the body weights of the experimental mice, and its effects were similar to those observed for the SASP-positive control group.

**Table 1:** Effects of CGMP on changes in the body weights of UC mice (%).

Group	n	Body weight changes in the 2nd day (%)	Body weight changes in the 3rd day (%)	Body weight changes in the 4th day (%)	Body weight changes in the 5th day (%)
N	20	+1.7±0.7	+2.9±1.2	+3.9±1.7	+5.1±1.3
M	20	-5.3±0.9**	-8.7±1.4**	-10.8±1.3**	-12.3±0.8**
C1	20	-3.9±0.5**	-3.5±1.3**	+0.9±1.3^##^	+3.1±2.7^##^
C2	20	-4.3±1.1**	-4.4±1.5**	-2.0±1.4^##^	+0.07±1.0^#^
S	20	-4.0±0.7**	-3.9±2.2**	-0.15±2.7^##^	+1.9±3.1^##^

Mice were treated with vehicle alone (50% ethanol, group N) or 1% famoxadone along with 50 mg/kg CGMP (group C1), 200 mg/kg CGMP (group C2), and 40 mg/kg SASP (group S). The mouse body weight was measured for 5-consecutive days following treatment.

* indicates a significant difference compared with the normal control group (P < 0.05)

** indicates an extremely significant difference compared with the normal control group (P < 0.01)

# indicates a significant difference compared with the model group (P < 0.05); and

## indicates an extremely significant difference compared with the model group (P < 0.001).

### Treatment with milk-derived CGMP reduced intestinal morphological and histological injury in experimental UC mice

The mouse intestinal morphological injury indices directly reflected the severity of injury and the degree of intestinal UC. Non-mucous hyperemia or ulcer formation was found in the mice in the normal control group, whereas distal intestinal mucous hyperemia, erosion and ulcer formation were observed in mice in the OXZ-induced UC model group. There were only a few small bloody spots in the distal intestinal mucosae, and the erosion and ulcer formation were significantly ameliorated in mice in CGMP group 1 and CGMP group 2 as well as in the SASP-treated group.

**Fig 1:**
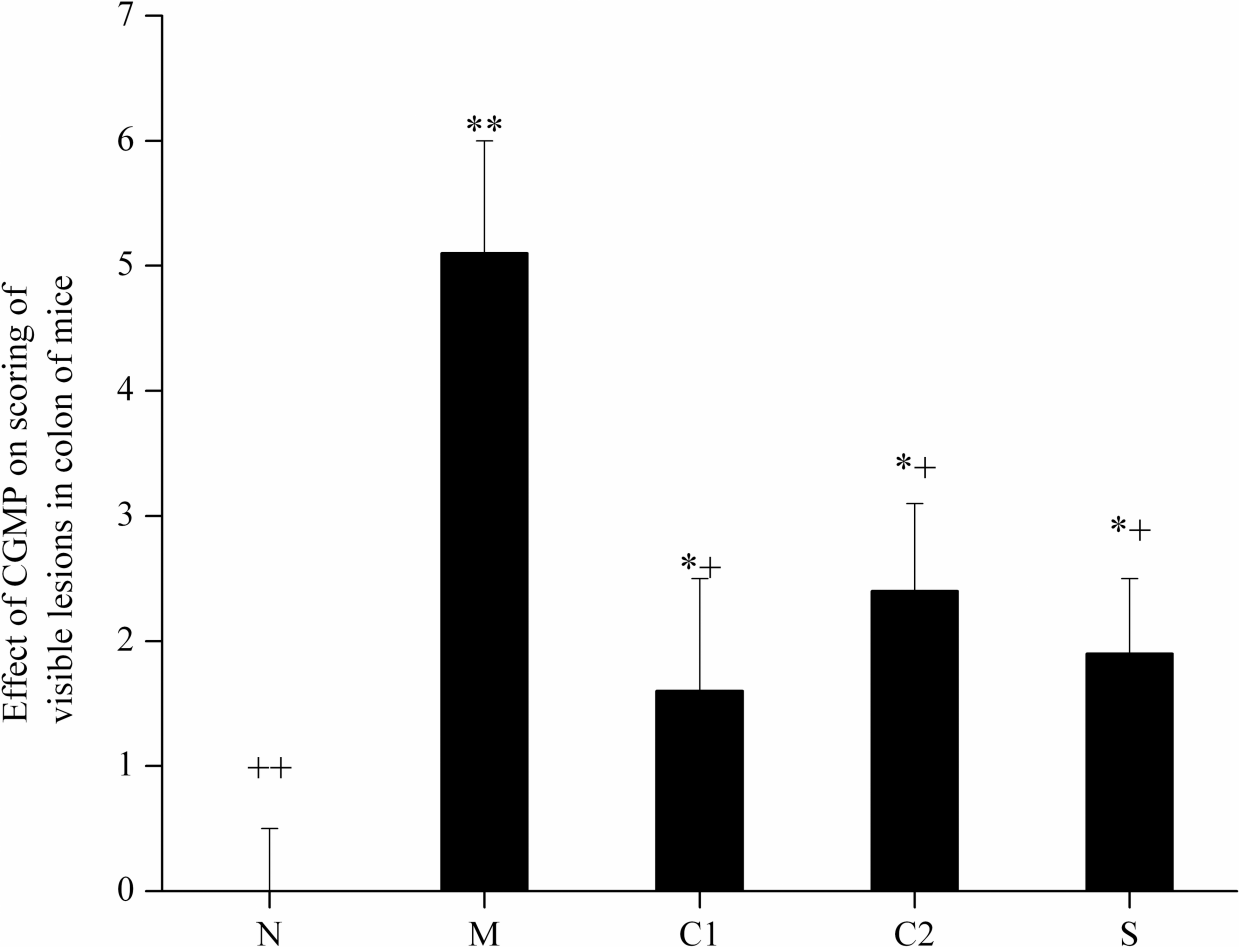
Effects of CGMP on mouse intestinal morphological injury index scoring. N: normal control group; M: OXZ-model group; S: SASP-treated group; C1: CGMP group 1 (50 mg/kg bw•d) and C2: CGMP group 2 (200 mg/kg bw•d). Each mouse in the five groups was anatomized: its abdominal cavity was opened, its intestines were separated and the stool was removed. Changes in intestinal morphology were observed, and pathologically altered sites were scored. The scoring criteria were divided into six grades according to the severity of injury in the intestinal mucosa. Grades above the 5th grade indicate two or more sites with serious ulceration and/or inflammation or one site where ulceration/inflammation was observed. When injured sections longer than 1 cm in the intestine on the vertical axis were observed, 1 scoring point was added; the highest scoring point was 10. Within the figure,* indicates a significant difference compared with the normal control group (P < 0.05); ** indicates an extremely significant difference compared with the normal control group (P < 0.01); + indicates a significant difference compared with the model control group (P < 0.05); and ++ indicates an extremely significant difference compared with the model control group (P < 0.01).

As shown in Fig 1, the scores for the intestinal morphological injury index for mice in the OXZ-induced UC model group, in which UC formed after enema administration, were significantly higher than those of the normal control group. Compared with the mice in the OXZ-model group, the intestinal morphological injury index scores for mice in CGMP group 1, CGMP group 2 and the SASP-treated group were significantly lower. The improved effects in mice from the CGMP group 1 and the SASP-treated group were most significant, but they were still higher compared than those of the normal control group. These results suggest that treatment with CGMP significantly improved intestinal morphological injury in experimental UC mice to a certain extent.

**Fig 2:**
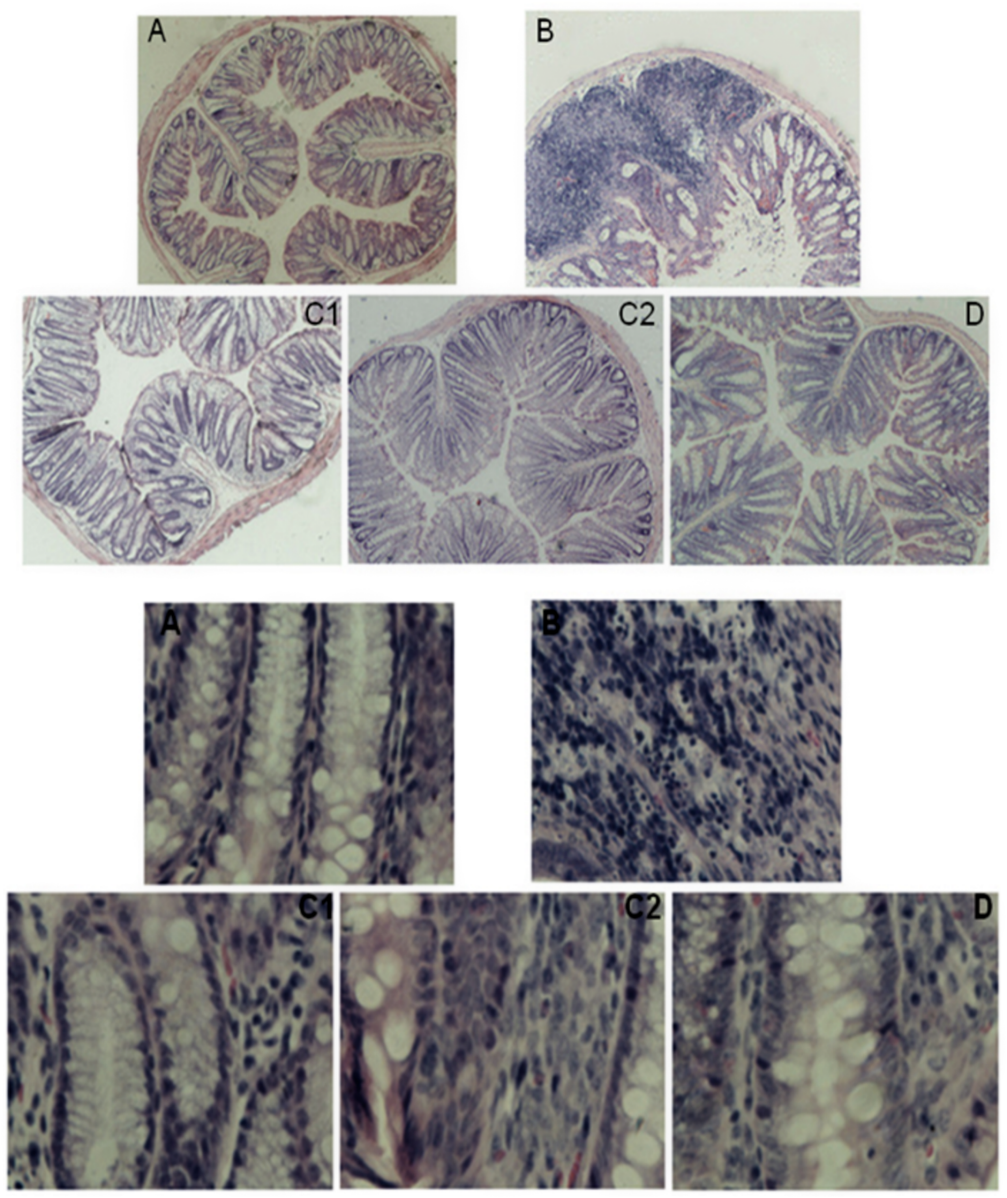
Images of intestinal histopathological sections from mice in the various groups: H&E staining with magnification at 40x and 400x. One-centimeter sections of normal and obviously pathologically altered intestine were removed from mice in each group, and paraffin sections were prepared using routine procedures, including paraffin embedding, sectioning, H&E staining and mounting with neutral balsam. Histopathological changes were observed with an inverted microscope. A: normal control group; B:OXZ-induced UC model group; C1: CGMP group 1 (50 mg/kg bw•d); C2: CGMP group 2 (200 mg/kg bw•d); and D: SASP-treated group. The upper image contains “A, B, C1,C2 and D” were based on images of H&E-stained sections at 40x magnification and The lower image contains bold “A, B, C1,C2 and D” were based on images of H&E-stained sections at 400x magnification.

**Fig 3:**
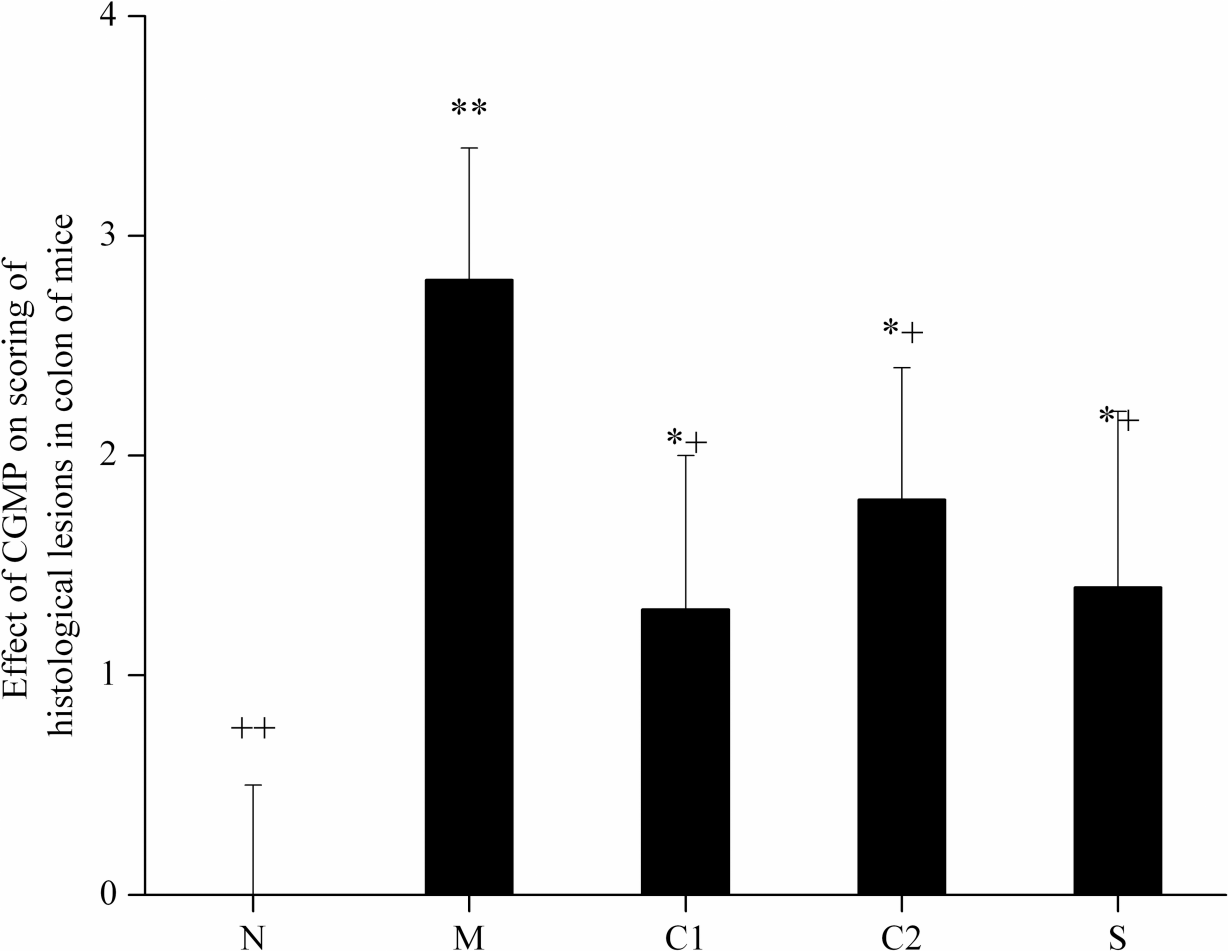
The effects of CGMP on mouse intestinal morphological and histological injury evaluation scores. Based on images of H&E-stained sections at 40x and 400x magnification, mouse intestinal morphological and histological injury were evaluated based on the status of crypt loss and the severity of erosion. The criteria were divided into 5 grades. Within the figure, * indicates a significant difference compared with the normal control group (P < 0.05); ** indicates an extremely significant difference compared with the normal control group (P < 0.01); + indicates a significant difference compared with the OXZ-model group (P < 0.05); and ++ indicates an extremely significant difference compared with the OXZ-model group (P < 0.01).

The mice in the various groups were anatomized on the 4^th^ day after enema. Their intestines were removed, and paraffin sections were prepared and visualized under a microscope. The intestinal epithelial mucosae of mice in the normal control group were intact, and the histology of the lamina propria of the intestinal mucosae was normal and arranged in an orderly manner (Figs 2A and 3A). No erosion or inflammatory cell infiltration were observed. For the mice in the OXZ-model group, their intestinal epithelial mucosae were injured and defective. The histology of the lamina propria mucosae was deformed, arranged in a disorderly manner and accompanied by erosion to a certain extent along with inflammatory cell infiltration (Figs 2B and 3B). For the mice in CGMP group 1, CGMP group 2 and the SASP-treated group, their intestinal epithelial mucosae were relatively intact, and the severity of intestinal mucosal injury was remarkably ameliorated (Figs 2C, 2D, 3C and 3D). The lamina propria of the intestinal mucosae began to return to normal conditions and exerted certain restorative effects on the intestinal mucosae.

As shown in Fig 3C1, the severity of intestinal tissue injury in the mice from the OXZ-induced UC model group was significantly increased compared with mice in the normal control group, whereas the severities of intestinal histological injury in the mice of CGMP group 1, CGMP group 2, and the SASP-treated group were significantly reduced compared with the mice in the OXZ-model group. These results suggested that treatment with CGMP significantly reduced intestinal histology injury in experimental UC mice.

### Regulation of CD4, CD8, MAdCAM-1 and sIgA expression in the intestinal mucosae of experimental UC mice who were treated with milk-derived CGMP

**Fig 4:**
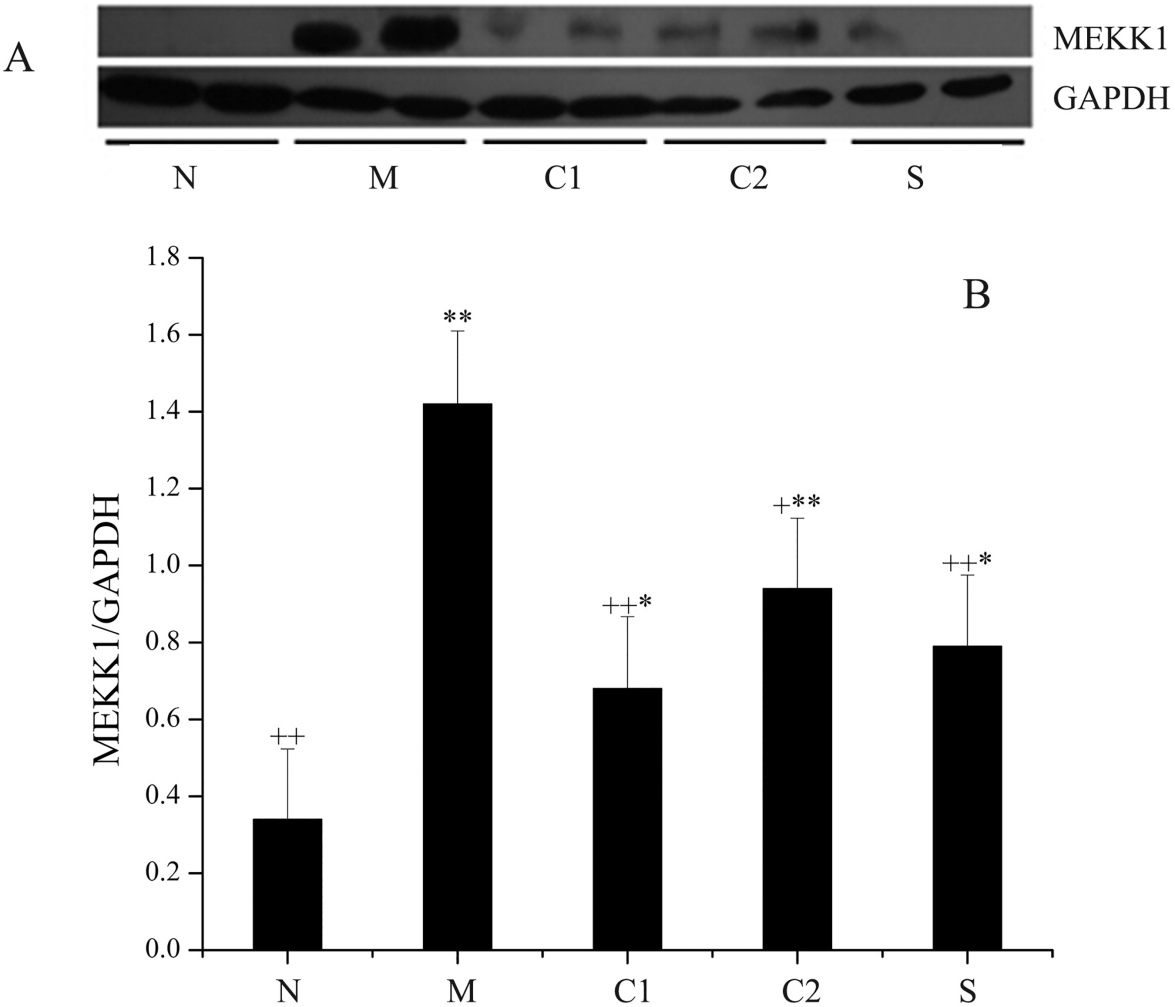
Effects of milk-derived CGMP treatment on the expression levels of CD4, CD8, MAdCAM-1 and sIgA in the mouse intestinal mucosa. After treatment with milk-derived CGMP at the indicated doses, the intestinal tissues of the mice in the various groups were removed and the expression levels of immunological barrier factors were detected via immunohistochemical staining methods. Fig 4A, 4B, 4C and 4D show the immunohistochemical staining results for CD4, CD8, MAdCAM-1 and sIgA, respectively. Within the figure, * indicates a significant difference compared with the normal control group (P<0.05); ** indicates an extremely significant difference compared with the normal control group (P < 0.01); + indicates a significant difference compared with the model control group (P < 0.05); and ++ indicates an extremely significant difference compared with the model control group (P < 0.01).

Identification of immunological barrier factors against UC is valuable and helpful to elucidate the molecular basis for the effects of milk-derived CGMP on the amelioration of symptoms and as a therapeutic treatment for UC. The CD4 molecule is primarily expressed by helper T cells (Th) and is the receptor for T cell receptor (TCR)-recognition of antigen in Th cells. CD4 binds to the non-polypeptide region of major histocompatibility complex (MHC) class II molecules and is primarily involved in TCR-mediated signal transduction in Th cells. CD8 is primarily expressed by cytotoxic T cells (Tc) and plays an important role in antigen recognition and presentation during specific immunological responses. Mucosal vascular addressin cell adhesion molecule 1 (MAdCAM-1) is an adhesion molecule that is selectively expressed on the surface of the intestinal mucosa and is related to endothelial cells of the lymphoid tissue. MAdCAM-1 is involved in mediating the directional homing of lymphoid cells toward the intestinal mucosa and thus is closely related to the initiation and development of UC. sIgA is a major immunoglobulin present in the intestinal mucosa and is the first line of defense to maintain intestinal mucosa homeostasis; thus, sIgA plays a defensive role in the intestine against symbiotic bacteria and invading pathogens. The results of immunohistochemical staining for sIgA in the intestinal tissues of mice from various groups are shown in Fig 4D. Considering the important role of CD4, CD8, MAdCAM-1 and sIgA in T cell responses and homing as well as mucosa homeostasis, we next sought to determine the effects of CGMP treatment on the expression levels of these molecules in experimental UC mice.

As shown in Fig 4A, the expression levels of CD4 were significantly increased in the lamina propria of the intestinal mucosae from mice of the OXZ-induced UC model group, and its IOD value was obviously higher than that of the normal control group. The difference between the two groups was extremely significant (*P* = 0.0043, *P* < 0.01). The expression levels of CD4 were significantly reduced in the lamina propria of the intestinal mucosae in the mice from CGMP Group 1, CGMP Group 2 and the SASP-treated group compared with the OXZ-model group. Of these, the differences between CGMP group 1 or the SASP-treated group and the OXZ-model group were extremely significant (*P* = 0.0061, *P* < 0.01), but they were still lower than the difference between the normal control group and the OXZ-model group, i.e., the CD4 expression levels in CGMP group 1 and the SASP-treated group were not restored to normal.

As shown in Fig 4B, the expression levels of CD8 were increased in the lamina propria of the intestinal mucosae in the mice of the OXZ-model group compared with the normal control group, and the difference between the two groups was statistically significant (*P* = 0.0250, *P* < 0.05). The expression levels of CD8 were reduced to a certain extent in the lamina propria of the intestinal mucosae in the mice of CGMP group 1, CGMP group 2 and the SASP-treated group compared with the OXZ-model group, but the differences among these groups were not statistically significant. The expression levels of CD8 in these groups were still significantly higher than in the normal control group (*P* = 0.0381, *P* < 0.05).

As shown in Fig 4C, compared with the normal control group, the expression levels of MAdCAM-1 were increased in the lamina propria of the intestinal mucosae in the mice of the OXZ-model group, and the difference between the two groups was extremely significant (*P* = 0.0044, *P* < 0.01). The expression levels of MAdCAM-1 were remarkably higher in the lamina propria of the intestinal mucosae in the mice of CGMP group 1, CGMP group 2 and the SASP-treated group than the OXZ-model group, and the differences were extremely significant (*P* = 0.0072, *P* < 0.01). The expression levels of MAdCAM-1 were still significantly higher in the mice from CGMP group 2 and the SASP-treated group than from the normal control group (*P* = 0.0288, *P* < 0.05), but no significant difference was observed between the CGMP group 1 and the normal control group.

As show in Fig 4D, compared with the normal control group, the expression levels of sIgA were significantly decreased in the lamina propria of the intestinal mucosae in the mice from the OXZ-model group, and the difference between the two groups was extremely significant (*P* = 0.0026, *P* < 0.01). The expression levels of sIgA were greatly increased in the lamina propria of the intestinal mucosae in the mice of CGMP group 1, CGMP group 2 and the SASP-treated group, and the difference among these groups was extremely significant (*P* = 0.0038, *P* < 0.01). The expression levels of sIgA were still significantly lower in CGMP group 2 than in the normal control group. However, the differences in the levels of sIgA for CGMP group 1 and the SASP-treated group were not significantly different from the control group, and the effects observed for the three groups were quite similar. Taken together, CGMP treatment downregulated expression of CD4, CD8 and MAdCAM-1 and upregulated expression of sIgA in the intestinal mucosae of UC mice.

### Regulation of MEKK1, Smad3, Smad7 expression in the intestinal mucosae of experimental UC mice who were treated with milk-derived CGMP

**Fig 5:**
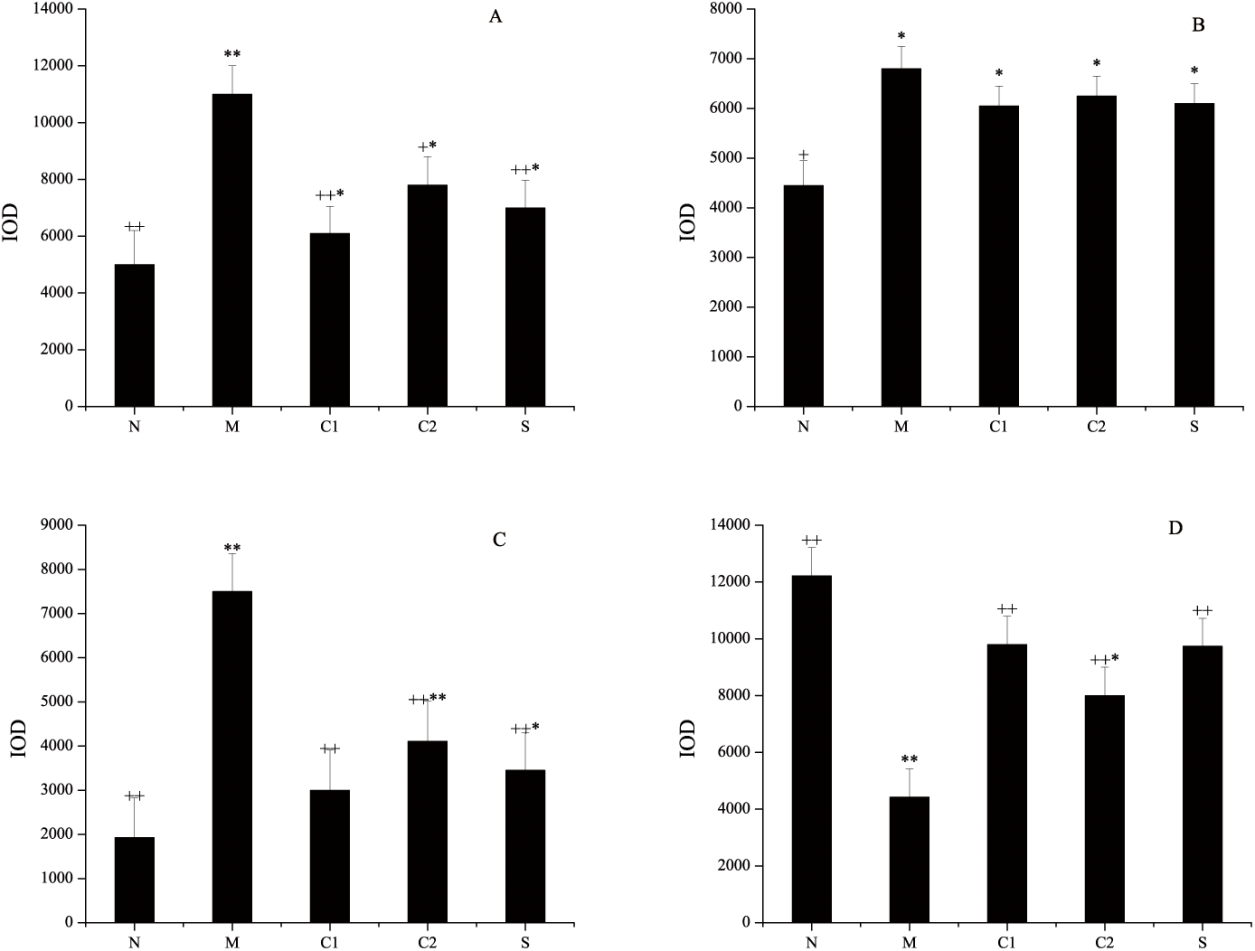
Expression levels of MEKK1 protein in the intestinal tissues of mice in the various groups. On the 4th day of treatment with milk-derived CGMP, pathologically altered intestinal sections were removed and weighed. Total protein was extracted from these samples, and the protein concentration was assayed using the BCA method. The expression levels of MEKK1 in the intestinal mucosae of UC mice in the various groups were detected by Western blotting, and the results are shown in Fig 5A. The gray integration values for the corresponding protein bands were acquired with Quantity One analysis software. GAPDH was used as the internal reference for quantitative normalization of the target protein MEKK1. The relative gray integration values for MEKK1 levels in various groups of samples are shown in Fig 5B. N: normal control group; M: OXZ-model group; S: SASP-treated group; C1: CGMP group 1 (50 mg/kg bw•d); and C2: CGMP group 2 (200 mg/kg bw•d).* Indicates a significant difference compared with the normal control group (P < 0.05); ** indicates an extremely significant difference compared with the normal control group (P < 0.01); + indicates a significant difference compared with the model control group (P < 0.05); and ++ indicates an extremely significant difference compared with the model control group (P < 0.01).

As seen in Fig 5, the expression level of MEKK1 in the OXZ-model group was extremely significantly increased compared with that of the normal control group (*P* = 0.0068, *P* < 0.01). The expression levels of MEKK1 in CGMP group 1, CGMP group 2 and the SASP-treated group were significantly lower than in the OXZ-model group, and the differences between these groups and the OXZ-model group were significant. Among these, the difference between CGMP group 1 and the OXZ-model group, as well as the difference between the SASP-treated group and the OXZ-model group, were extremely significant (*P* = 0.0088, *P* < 0.01). However, the expression levels of MEKK1 in these two groups were still significantly higher than in the normal control group.

Smad proteins are the only substrates for the receptors that trigger the TGF-β1 signaling pathway. When organisms are stimulated by harmful factors, TGF-β1 binds to the serine-threonine kinase complex, which in turn triggers the transduction of this signaling pathway. Among the members of the pathway, Smad-7 is a major negative regulatory protein. An abnormal increase in Smad-7 expression levels may be the major cause of the TGF-β1-mediated pathogenesis of IBD.

**Fig 6:**
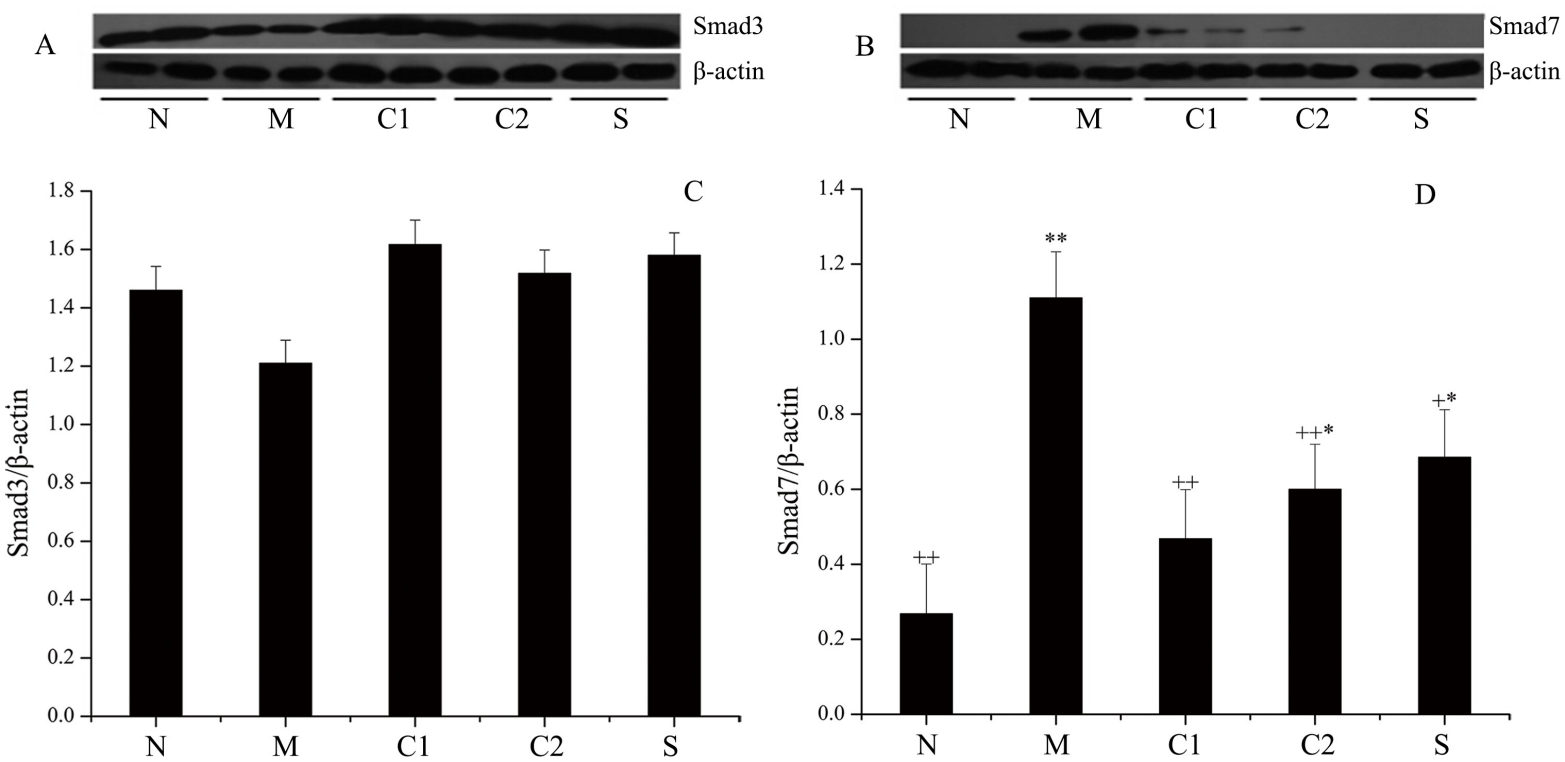
Expression levels of Smad-3 and Smad-7 in the intestinal tissues of mice in the various groups. The expression levels of Smad-3 and Smad-7 in the intestinal mucosae of UC mice in the various groups were detected by Western blotting, and the results are shown in Fig 6A and 6B. The gray integration values for Smad-3 and Smad-7 protein bands were acquired with Quantity One analysis software. β-actin was used as the internal reference for quantitative normalization of the target proteins, Smad-3 and Smad-7, and relative gray integration values for Smad 3 and Smad 7 levels in various sample groups were obtained and showed in Fig 6C and 6D. N: normal control group; M: OXZ-model group; S: SASP-treated group; C1: CGMP group 1 (50 mg/kg bw•d); and C2: CGMP group 2 (200 mg/kg bw•d). * Indicates a significant difference compared with the normal control group (P < 0.05); ** indicates an extremely significant difference compared with the normal control group (P < 0.01); + indicates a significant difference compared with the model control group (P < 0.05); and ** indicates an extremely significant difference compared with the model control group (P < 0.01).

As shown in Fig 6, the expression levels of Smad-3 in the intestines of mice from the OXZ-model group were reduced compared with the normal control group, but the difference between the two groups was not statistically significant (*P* = 0.1738, *P* > 0.05). The expression levels of Smad-3 in the mice from CGMP group 1, CGMP group 2 and the SASP-treated group were increased compared with that of the mice in the OXZ-model group, but the differences was not significant, i.e., the effects of GCMP and SASP on the expression of Smad 3 were not statistically significant (*P* = 0.1925, *P* >0.05). However, the expression levels of Smad-7 in the intestines of the mice from the OXZ-model group were markedly increased compared with the normal control group, and the difference was extremely significant (*P* = 0.0016, *P* < 0.01). The expression levels of Smad-7 in the mice from CGMP group 1, CGMP group 2 and the SASP-treated group were reduced compared with that of the mice in the OXZ-model group, and the differences between CGMP group 1 and the OXZ-model group and the differences between SASP group 2 and the OXZ-model group were extremely significant (*P* = 0.0034, *P* < 0.01). The difference between CGMP group 2 and the SASP-model group was also significant (*P* = 0.0145, *P* < 0.05), but the difference between CGMP group 1 and the OXZ-model group was not significant, indicating that treatment of UC mice with CGMP at 50 mg/kg bw•d caused optimal effects in terms of reduced OXZ-induced expression of Smad-7. Collectively, these results indicate that treatment with CGMP significantly inhibits the MAPK signaling pathway and activates the TGF-β1/Smad signal transduction cascade in the intestinal mucosae of UC mice.

## Discussion

Ulcerative colitis is a chronic, non-specific inflammatory disorder in the rectum and colon, and its associated pathological changes primarily occur in the epithelial layer of the intestinal mucosa. It has been found that multiple factors, including genetic factors, immunological factors, cytokines, inflammatory mediators, and environment, are all involved in UC pathogenesis [28]. Thus, UC exhibits multiple clinical symptoms and easily recurs with increasing complications [12]. To date, the symptoms of UC can only be ameliorated, and there are no therapeutic approaches available for the effective and complete cure of UC. In this study, we used 3% OXZ to induce mouse skin sensitization and conducted a direct enema with 1% OXZ to establish a mouse model of UC, which was confirmed by observing changes in the general state of the mice as well as evaluating scores for the morphological injury index and histology and morphological observation as previously described[23, 24]. Then, we intragastrically administered CGMP to OXZ-induced UC mice at 50 mg/kg bw•d, and observed intestinal loss and pathological changes, we confirmed that CGMP could ameliorate UC symptoms, exert certain roles in repair and restoration following injury to the intestinal mucosa and maintain intestinal tissue integrity. These beneficial effects are similar to those of SASP, a medicine clinically used for the treatment of IBD.

CGMP was administered to experimental UC mice in two different dosages (50 and 200 mg/kg bw•d). However, a dose-dependent relationship was not observed at these amounts. It appeared that the typical 200 mg/kg bw•d dosage would be too high and the 50 mg/kg bw•d dose group had a more significant difference compared with the control group. The reason for this observation may be that dosing of CGMP in mice is not linear as the intestine may only be able to absorb a limited amount of CGMP. We need further experiments to explore dose dependence and whether dose suppression exists in experimental UC mice intragastrically administered CGMP. Additionally, the sensitivity of intestinal epithelial cells to CGMP and the mechanism of its absorption need further study. Currently, a few studies have been conducted to examine CGMP as a therapeutic treatment for UC. For example, the study conducted by Requena [29] found that bovine glycomacropeptide (BGMP) has an anti-inflammatory activity in the ileum with similar efficacy to 5-aminosalicylic acid by regulating the secretion of TNF-α, IL-1β, and IL-8 from monocytes and activating the NF-κB and MAPK signal transduction pathways. Daddaoua [30] also investigated the therapeutic effects of CGMP on the 3-nitrobenzenesulfonic acid (TNBS)-induced UC mice model and found that intragastric administration of CGMP at appropriate doses could effectively reduce morphological injury and the expression of inflammatory factors such as inducible nitrite oxide synthase (iNOS) and activator protein (AP) in experimental UC mice. Requena [31] studied the therapeutic effects of CGMP on TNBS-induced ileitis in mice and confirmed that intragastric administration of CGMP in these mice at appropriate doses could significantly reduce morphological injury, myeloperoxidase (MPO) activity, and the expression levels of iNOS and cyclooxygenase-2 (COX-2), and the anti-inflammatory effects of CGMP were the same as those of 5-aminosalicylic acid, a medicine used to therapeutically treat IBD.

The intestinal mucosal barrier is an important component of an organism’s barrier system, which primarily consists of the intestinal mucosal mechanical barrier, chemical barriers, biological barriers and immunological barriers. These barriers have different structures, molecular regulatory mechanisms and biological functions. They also cooperate closely through their own signal transduction pathways to defend against the invasion of exogenous antigenic substances [32]. The dysfunction of the intestinal mucosal barrier may destroy the homeostasis of the internal environment within the body and cause systemic inflammatory responses [33]. Thus, studying the dysfunction of the intestinal mucosal barrier will not only elucidate the molecular basis underlying the dysfunction of the intestinal mucosal barrier but will also facilitate the control and restoration of intestine-related diseases. In the present study, we conducted an investigation of the role of CGMP in the regulation of key functional factors in the intestinal mucosa and revealed that CGMP could restore the intestinal health of experimental UC mice by regulating the function of the intestinal mucosal barrier.

It has been confirmed that MAdCAM-1 is an important target for the therapeutic treatment of chronic inflammatory bowel disease (IBD) [34]. MAdCAM-1 interacts with α4β7 integrin to mediate the entry of intestinal mucosal lymph cells into inflammatory sites, where they are involved in inflammatory responses, inducing chronic inflammation. The expression levels of MAdCAM-1 were increased at inflammatory sites in UC patients [35, 36]. The results of this study have shown that CGMP is capable of significantly downregulating the expression levels of MAdCAM-1 in the lamina propria of the intestinal mucosa in OXZ-induced UC mice, indicating that MAdCAM-1 plays an important role in the amelioration of functional disorder and protection of the intestinal mucosal barrier.

Based on the differential expression of specific antigen receptors on their surfaces, T lymphocyte cells (T cells) can be divided into two classes: CD4^+^ T cells and CD8^+^ T cells. Both CD4^+^ T cells and CD8^+^ T cells are the lynchpins of immunological regulation, and together they control the intensity of immune responses. The ratio of CD4^+^ T cells/CD8^+^ T cells is an important indicator reflecting the functional status of T-lymphocyte cells [37]. Disproportionate T cell subpopulations, imbalanced immunological regulation, and abnormal expression and function of cytokines and other cellular receptors can all lead to aberrations in inflammatory cells and inflammatory mediators, which in turn cause injury to the intestinal mucosa. Thus, both CD4 and CD8 play important roles in the amelioration and treatment of UC. Brandhorst et al. [38] stated that CD4^+^-mediated immune responses were a potential biomarker for IBD inflammatory responses because CD4^+^-mediated immune responses in patients with IBD were significantly enhanced. A study by Li et al. [39] demonstrated that the infiltration of a large number of T-lymphocyte cells into the lamina propria of the intestinal mucosae of patients with UC. The expression levels of CD4 and CD8 were significantly increased, and the ratio of CD4/CD8 was highest during the active period of UC. The abnormal expression of CD4 and CD8 led to a loss of control in terms of acquired immunity in UC patients. The results of this study showed that, compared with the normal control group, the increase in the expression levels of CD4 in the lamina propriety of the intestinal mucosae of the mice in the OXZ-induced UC model was more significant than the increase in the expression levels of CD8; thus, this led to an increased ratio of CD4^+^/CD8^+^ T cells in the lamina propria of the intestinal mucosae of these mice. These experiments indicated that the increased expression levels of CD4 in the OXZ-induced UC model will strengthen the functions of CD4^+^ T lymphocyte cells and increase the release of a large amount of proinflammatory factors to stimulate the activation of B-lymphocyte cells to generate antibodies and cause hyperresponses via humoral immunity. This activity further stimulates the complement system, which in turn triggers inflammatory responses in the intestinal mucosa. The findings in this study are consistent with those reported by Neurite [40], which showed that CD4^+^ T lymphocyte cells help to trigger the initiation and maintenance of inflammation in the intestine. This study has further revealed that CGMP can act similarly to SASP and reduce the expression levels of CD4 and CD8 and, in particular, cause a significant reduction in CD4, causing immunological elimination and immunological tolerance to be maintained in a balanced state and stimulating repair and restoration after inflammation and injury.

Intestinally secreted IgA (sIgA) is the first line of defense to maintain homeostasis of the intestinal mucosa. It plays an important role in immune regulation by protecting the integrity of the intestinal biological barrier. As an important protein factor, sIgA has received widespread attention from a number of investigators [41]. For example, Li et al. [42] found that the expression levels of sIgA in UC patients were lower than in normal controls, but after treatment with bifidobacterium, the expression levels of sIgA significantly increased again. Elvira et al. [43] found that in a DSS-induced UC model mice, increased levels of IgG and reduced levels of sIgA followed the progression of UC, and the levels of sIgA were significantly downregulated in the intestinal tissues of experimental UC mice, leading to serious injury to the intestinal tissue. Furthermore, another study demonstrated that the anti-inflammatory effects of sIgA were mediated by inhibited production of lipopolysaccharide (LPS)-induced cytokines, such as TNF-α and IL-1β [44]. Additionally, Brandtzaeg et al.[45] noted that human IgA immune deficiency was closely correlated with UC. The results of this study indicated that the expression levels of sIgA in the lamina propria of the intestinal mucosae of OXZ-induced UC mice were significantly reduced. After treatment with milk-derived CGMP, the expression levels of sIgA in the lamina propria of the intestinal mucosae were significantly increased, and the stimulatory effects were similar to the improved effects induced by SASP, a medicine used to treat IBD. Thus, CGMP plays a very important role in immunological regulation through the stimulation of sIgA expression in the intestinal mucosa, the prevention of intestinal mucosal injury, and improvement of intestinal mucosal barrier function.

Natural correlations between the expression levels of cytokines/related proteins and the activation of specific immunological signaling pathways have been demonstrated. The major signaling pathways that are known to be closely related to the initiation and development of inflammation include NF-κB, MAPK and TGF-β [46]. Our previous studies confirmed that the expression levels of p65 in the NF-κB pathway are closely correlated with the development of UC [18, 27]. This study revealed that after intragastric administration of CGMP, both mRNA and protein levels of NF-κB p65 in inflammatory sites of the intestinal mucosae in OXZ-induced UC mice were significantly reduced compared with the normal control group. This observation indicated that the expression levels of NF-κB p65 play a key role in the development of UC inflammation and implied that CGMP may act as a negative regulator of NF-κB activities. Based on these results, we conducted further experiments to focus on how MEKK1 in MAPK plays a role in the initiation and development of intestinal UC and how its expression is correlated with activation of the NF-κB pathway. The study results indicated that the expression levels of MEKK1 in the intestinal mucosae of mice in the UC model group were significantly increased compared with the normal control group. This showed that the MAPK signaling pathway in the intestinal mucosal of experimental UC mice is activated, whereas intragastric administration of CGMP at 50 mg/kg bw•d significantly reduced the expression of MEKK1, and this inhibitory effect was similar to that of SASP, a medicine used to treat IBD. Therefore, the therapeutic effects of CGMP are mediated through its inhibition of MEKK1 expression and its direct involvement in the inhibition of NF-κB activation as well as its indirect inhibition of IKK-mediated IκB phosphorylation, which finally leads to the inhibition of NF-κB activation and control of inflammation. The results of this study are in agreement with those reported by Requena et al. [31] and Daddaoua [30] and enrich the research literature in term of the roles of CGMP in the opsonization, control and restoration of function during UC.

TGF-β1 is a multifunctional cytokine that regulates the growth, differentiation and function of multiple immunological and non-immunological cells. Both *in vitro* and *in vivo* studies have demonstrated that TGF-β1 can exert significant negative regulatory effects on inflammation in the intestinal mucosa[47]. Smad proteins are key players in the TGF-β1 signaling pathway in cells. A number of studies have found that the appearance and/or disappearance of intestinal UC inflammation may be related to Smad protein abnormalities during the TGF-β1 signal transduction process [48]. Smad-3 is a receptor-regulatory type Smad. A previous study reported that Smad-3 was involved in TGF-β1-mediated inhibition of the activation and proliferation of T cells and that decreases in the expression levels of Smad3 could reduce T cell responses to TGF-β1 [49, 50]. Smad-3 knockout mice died 6 months after birth due to deficiencies in their intestinal immunological functions. These mutant mice lost their ability to respond to TGF-β1, and the infiltration of large numbers of T cells in the intestine were observed [51]. Ulloa et al. [52] reported that IFN-γ inhibited the TGF-β1-induced phosphorylation of Smad-3, which in turn inhibited the association of Smad-3 with Smad-4 and retarded the activation of TGF-β1. A study by Feinberg et al. [53] confirmed the inhibition of the protein expression levels of MCP-1, a chemokine secreted by mononuclear cells that plays a role in the recruitment of macrophages during inflammation. Thus, Smad-3 exerts its anti-inflammatory role via its phosphorylation to trigger the TGF-β1 signaling pathway. However, our study results indicated that the protein expression levels of Smad-3 in the intestinal mucosae of experimental UC model mice were reduced but were not significantly different from that of the normal control group (i.e., there was no significant difference in the protein expression levels of Smad-3 between the experimental UC model group and the normal control group) (*P*>0.05). However, treatment with CGMP or SASP induced increased protein expression levels of Smad-3 in the intestinal mucosae of mice, although the stimulatory effects were not significant compared with that of the OXZ-model group. Both CGMP and SASP modulated the TGF-β1 signaling pathway via regulatory Smad signal transduction and phosphorylation activation. The Smad-3 identified in this study was the inactive form of Smad-3. Thus, it can be inferred that the anti-inflammatory effects of nonphosphorylated Smad-3 are relatively weak and that Smad-3 must first be activated by phosphorylation to better perform its anti-inflammatory roles and mediate the TGF-β1 signaling pathway. Thus, the mechanisms by which the nonphosphorylated and phosphorylated forms of Smad-3 are involved in the initiation and development of intestinal UC require further study.

Smad-7 is an inhibitory type Smad that inhibits the TGF-β1 signaling pathway and retards the phosphorylation of receptor-type Smads. The enhanced expression of Smad 7 can restrict the amplification of the TGF-β1/Smad signaling cascade and thus inhibit the anti-inflammatory functions of TGF-β1 in UC and stimulate the restoration of the intestinal mucosa [54]. The results of this study indicated that the protein expression levels of Smad7 in the intestine of the mice in the experimental UC model group were significantly increased compared with the normal control group, and the difference between the two groups was extremely significant (*P*<0.01), whereas treatment of experimental UC mice with CGMP at an appropriate dose significantly reduced the protein expression levels of Smad7, and this inhibitory effect was the same as that caused by SASP. The inhibition of Smad-7 expression can restore the anti-inflammatory effects of TGF-β1 [53]. Additionally, intestinal TGF-β1 plays a role in the negative regulation of NF-κB activation, whereas Smad7 inhibits the activation of the NF-κB pathway by retarding the TGF-β1 signaling pathway during intestinal inflammatory responses. Therefore, based on results described above, it is clear that milk-derived CGMP can inhibit the expression of the Smad7 protein, directly activate the amplification of the TGF-β1/Smad signal cascade and indirectly activate the activity of IκBα and cooperatively inhibit the activation of NF-κB, thus maintaining the immunoregulatory balance and protecting the functions of the immunological barrier, which effectively exert an anti-inflammatory role in the intestinal mucosa. To date, there have been no detailed reports regarding the involvement of CGMP in the regulation of the TGF-β1/Smad signaling cascade, which is an important signaling pathway that is closely related to IBD. In this study, by investigating the effects of CGMP on the expression levels of Smad3 and Smad7, two important regulatory proteins involved this signaling cascade, we have confirmed that CGMP is involved in the regulation of the TGF-β1/Smads signaling cascade, which intersects with the MAPK and NF-κB signaling pathways to form a complex signal network. These pathways communicate and interconnect with each other to maintain intestinal mucosa homeostasis, ameliorate UC symptoms and effectively control inflammatory responses.

## Conclusions

In this study, we found that milk-derived CGMP possesses the ability to significantly regulate and improve UC by reducing the expression levels of MAdCAM-1, CD4 and CD8 and by stimulating the expression of sIgA in the lamina propria in the intestinal mucosa. Milk-derived CGMP is directly involved in inhibiting the activation of MAPK and inactivating the TGF-β1/Smad cascade, maintaining the balance of immunological regulation in the intestinal mucosa and protecting the functions of the intestinal mucosal barrier via effective inhibition of the expression of the MEKK1 and Smad7 proteins, two key proteins that are involved in the MAPK and TGF-β1 signaling pathways. Therefore, this study has revealed that, at the molecular level, milk-derived CGMP plays an important role in maintaining homeostasis of the internal environment in the intestines of experimental UC mice, thus ameliorating the pathological symptoms of UC and significantly controlling the initiation and development of inflammatory responses within the body.
